# Amyloid assembly and disassembly

**DOI:** 10.1242/jcs.189928

**Published:** 2018-04-13

**Authors:** Edward Chuang, Acacia M. Hori, Christina D. Hesketh, James Shorter

**Affiliations:** 1 Department of Biochemistry and Biophysics, Perelman School of Medicine at the University of Pennsylvania, Philadelphia, PA 19104, USA; 2 Pharmacology Graduate Group, Perelman School of Medicine at the University of Pennsylvania, Philadelphia, PA 19104, USA

**Keywords:** Amyloid, Autophagy, Disaggregase, Prion, Neurodegeneration

## Abstract

Amyloid fibrils are protein homopolymers that adopt diverse cross-β conformations. Some amyloid fibrils are associated with the pathogenesis of devastating neurodegenerative disorders, including Alzheimer's disease and Parkinson's disease. Conversely, functional amyloids play beneficial roles in melanosome biogenesis, long-term memory formation and release of peptide hormones. Here, we showcase advances in our understanding of amyloid assembly and structure, and how distinct amyloid strains formed by the same protein can cause distinct neurodegenerative diseases. We discuss how mutant steric zippers promote deleterious amyloidogenesis and aberrant liquid-to-gel phase transitions. We also highlight effective strategies to combat amyloidogenesis and related toxicity, including: (1) small-molecule drugs (e.g. tafamidis) to inhibit amyloid formation or (2) stimulate amyloid degradation by the proteasome and autophagy, and (3) protein disaggregases that disassemble toxic amyloid and soluble oligomers. We anticipate that these advances will inspire therapeutics for several fatal neurodegenerative diseases.

## Abstract (precis)

**Summary:** This Review showcases important advances in our understanding of amyloid structure, assembly and disassembly, which are inspiring novel therapeutic strategies for amyloid disorders.

## Introduction

**Fig. 1.**
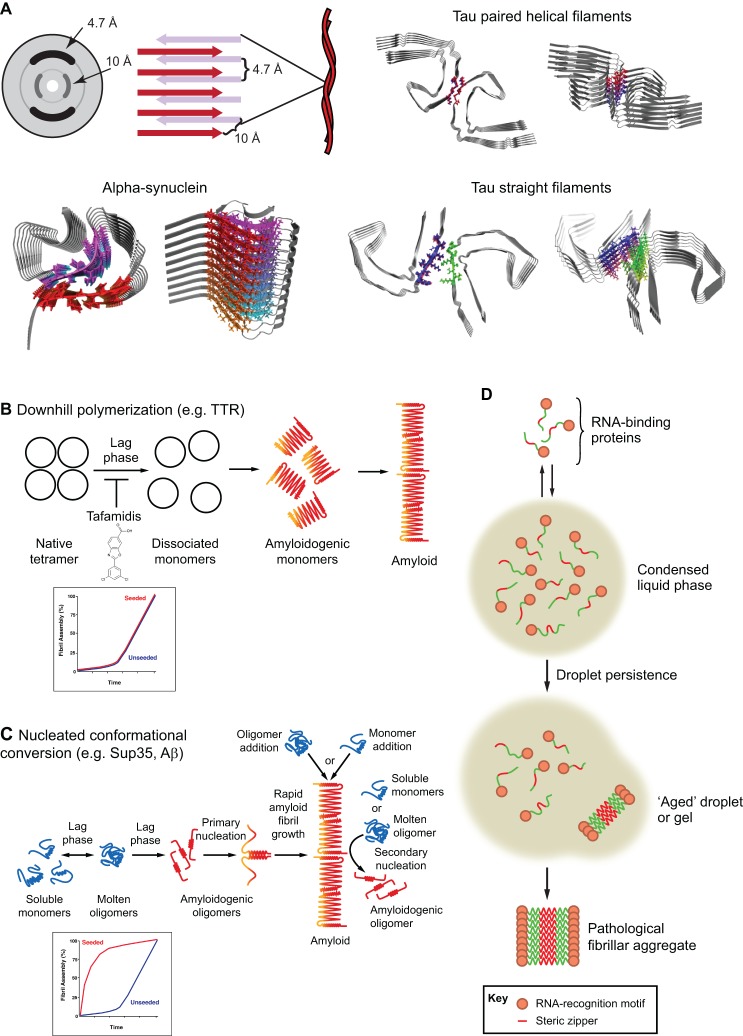
**Amyloid structure and formation pathways.** (A) Top-left: the X-ray diffraction pattern for amyloids shows major reflections at ∼4.7 Å (hydrogen bonding distances between β-strands) and ∼10 Å (side-chain packing between β-sheets) indicating cross-β structure where β-strands align perpendicular to the fibril axis. Bottom-left: solid-state NMR structure of human α-synuclein fibril (PDB: 2N0A) (Tuttle et al., 2016). Right side: 3.4 Å–3.5 Å resolution cryo-EM structures of tau paired-helical filaments (PDB: 5O3L) and straight filaments (PDB: 5O3T) from an AD patient (Fitzpatrick et al., 2017). (B) In downhill polymerization (DP), the lag phase of amyloid formation is due to the slow dissociation of a stable native tetramer into monomers, which then rapidly assume an amyloidogenic conformation. This mechanism is employed by TTR in FAP (Hurshman et al., 2004). TTR amyloidosis can be inhibited by tafamidis, a drug that stabilizes TTR in its native tetrameric state (Coelho et al., 2012). Thus, understanding the mechanism of amyloid formation can enable development of drugs to preserve the native state and prevent amyloidogenesis. Typically, amyloids formed by DP do not eliminate the lag phase of fibrillization in reactions seeded with preformed fibrils (lower panel). (C) In nucleated conformational conversion (NCC), partially or completely disordered soluble monomers are initially in equilibrium with molten soluble oligomers. During the lag phase of assembly, these molten soluble oligomers gradually rearrange into amyloidogenic oligomers, which then rapidly form cross-β nuclei (primary nucleation), thereby ending the lag phase. As soon as cross-β nuclei have formed, fibrillization proceeds rapidly as nuclei recruit and convert soluble monomers and molten soluble oligomers into the cross-β form at the growing fibril ends. The introduction of pre-formed fibrils eliminates the lag phase of assembly via immediate templating of the amyloid conformation. The lateral face of the assembled fibril also serves as a site for secondary nucleation events where molten oligomers or soluble monomers can rapidly convert into amyloidogenic oligomers. Typically, amyloids formed by NCC eliminate the lag phase of fibrillization in reactions seeded with preformed fibrils (lower panel). (D) Phase transition of proteins containing prion-like domains (PrLDs). RBPs can condense into liquid droplets through transient interactions between PrLDs and other multivalent interactions. Droplet persistence enables formation of stable (less dynamic) interactions between PrLDs that drive an aberrant phase transition from liquid to solid states that comprise pathological fibrils, which accumulate in disease.

Amyloid fibrils are protein homopolymers that adopt diverse cross-β conformations (Fig. 1A). These non-branching fibrils are stabilized via intermolecular contacts between β-strands, which align orthogonally to the fibril axis to yield cross-β architecture (Fig. 1A) (Eanes and Glenner, 1968; Sipe and Cohen, 2000; Sunde et al., 1997). Amyloid is among the most stable protein conformations (Smith et al., 2006). Indeed, insulin amyloids have a strength of ∼0.6±0.4 GPa, which is comparable to that shown by steel (∼0.6–1.8 GPa) (Knowles and Buehler, 2011; Smith et al., 2006).

Amyloid fibrils occur naturally and perform specialized functions, including pigment formation, long-term potentiation (LTP), sperm selection and peptide hormone release (Box 1) (Berson et al., 2003; Drisaldi et al., 2015; Fioriti et al., 2015; Fowler et al., 2006; Maji et al., 2009; Pavlopoulos et al., 2011; Roan et al., 2017; Stephan et al., 2015; Watt et al., 2009). However, many proteins form amyloid fibrils that perturb cellular processes and underlie fatal neurodegenerative disorders and systemic amyloidoses (Blancas-Mejía and Ramirez-Alvarado, 2013; Guo and Lee, 2014).

Box 1. Functional extracellular amyloids in humans. Functional amyloid fibrils are naturally abundant in human semen. Indeed, low levels of seminal amyloid is correlated with reduced male fertility (Castellano and Shorter, 2012). Several fragments of prostatic acid phosphatase (PAP, also known as ACPP), such as PAP 248–286, PAP 85–120, and similarly, fragments of semenogelin 1 and 2 (SEM1 and SEM2) form amyloid fibrils in human seminal fluid (Bergman et al., 2016; Castellano and Shorter, 2012). These fibrils may have antimicrobial functions (Easterhoff et al., 2013; Usmani et al., 2014), protect sperm cells and serve as a filter that retains sperm of low quality, permitting only the fittest sperm to escape and fertilize the oocyte (Bergman et al., 2016; Castellano and Shorter, 2012; Roan et al., 2017). Unfortunately, these fibrils also promote HIV infection by several orders of magnitude (Arnold et al., 2012; Münch et al., 2007; Roan et al., 2011). Thus, agents that disrupt semen amyloid may reduce sexual HIV transmission. Notably, two small molecules, EGCG (a green tea polyphenol) and CLR01 (a lysine- and arginine-specific molecular tweezer) can remodel seminal amyloid and prevent HIV infection (Castellano et al., 2015b; Lump et al., 2015). Likewise, the protein disaggregase Hsp104, can be retooled to remodel and clear seminal amyloids and counter HIV infection (Castellano et al., 2015a).

**Fig. 2.**
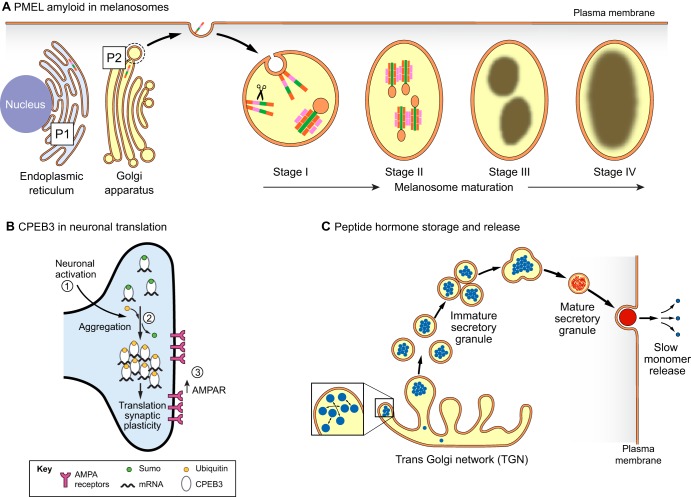
**Functional amyloids.** (A) PMEL forms functional amyloid in melanin metabolism. PMEL fibril formation is highly regulated by post-translational cleavage into its amyloidgenic form and compartmentalization within melanosomes during melanosome maturation. PMEL fibrils catalyze the formation of melanin, concentrate melanin and facilitate bulk transport of melanin (Watt et al., 2013). (B) CPEB3 is a regulator of mRNA translation in neurons and enhances LTP through positive regulation of AMPA receptor translation. CPEB3 is soluble and SUMOylated in its basal state. Upon neuronal activation, CPEB3 is deSUMOylated and ubiquitylated, causing the protein to aggregate and activate translation of certain mRNAs (Drisaldi et al., 2015). (C) Peptide hormones (blue) are concentrated in secretory granules where they form amyloids (red) as a packaging mechanism. Some peptide hormones aggregate spontaneously, while others require the assistance of glycosaminoglycans (Maji et al., 2009). Furthermore, these amyloid fibrils slowly depolymerize spontaneously upon vesicle release into the extracellular space, resulting in delayed release of monomeric hormones.

Various peptide and protein hormones are expressed as prohormones that are proteolytically processed and concentrated in secretory granules (Fig. 2C) (Goetze et al., 2012). Many of these hormones form amyloid fibrils *in vitro* and *in vivo* (Maji et al., 2009). Some hormones can form amyloid *in vitro* at the secretory granule pH of 5.5, but many require the assistance of glycosaminoglycans (GAGs) such as heparin to form amyloid (Maji et al., 2009). These hormones can be stored at high concentrations in the amyloid state, which enables delayed release of hormones as the fibrils slowly dissociate after secretion and degranulation (Fig. 2C). Hormone amyloids are often non-toxic, but some can be as neurotoxic as Aβ (Maji et al., 2009). However, they are not toxic when restricted to secretory granules. Assembly and disassembly rates of amyloid hormones are highly dependent on their storage and release environments (Jacob et al., 2016; Nespovitaya et al., 2016; Skeby et al., 2016). Specific factors such as pH, salt and GAGs tightly regulate peptide hormone amyloidogenesis, suggesting that degranulation or mislocalization drastically alters aggregation kinetics (Jacob et al., 2016; Nespovitaya et al., 2016; Skeby et al., 2016). Thus, amyloid can serve as a storage depot that slowly releases functional hormones after secretion (Fig. 2C).

The mechanisms of toxicity in amyloidoses are debated. One view is that amyloid fibrils, their soluble misfolded oligomeric antecedents or both are directly toxic to cells leading to a gain-of-toxicity phenotype (Bucciantini et al., 2002; Guo and Lee, 2014; Kayed et al., 2003; Olzscha et al., 2011). Another view is that the conversion of native proteins into misfolded conformations, including amyloid and soluble misfolded oligomers, results in a loss-of-function phenotype. Indeed, aggregation-prone proteins such as TDP-43 (encoded by *TARDBP*) that are involved in human disease can have essential functions (Harrison and Shorter, 2017; Lee et al., 2011a; Ward et al., 2014). These two mechanisms are not mutually exclusive and may synergize in some diseases (Harrison and Shorter, 2017). However, synthetically engineered amyloids or soluble misfolded oligomers with no native function can induce cell death and directly disrupt proteostasis (Bucciantini et al., 2002; Olzscha et al., 2011). Thus, there are likely universal gain-of-toxicity mechanisms induced by amyloid fibrils or soluble misfolded oligomers, which may be exacerbated by the loss of native protein function. While this generic toxicity unleashes havoc in the context of disease, nature has also quenched this toxicity and deployed amyloid for functional purposes (Bergman et al., 2016; Harvey et al., 2017; Hufnagel et al., 2013; Jarosz and Khurana, 2017; Watt et al., 2013). On the other hand, nature has also tuned amyloid-like structures to be highly toxic as with the remarkable cross-α fibrils formed by the phenol-soluble modulin α3 peptide secreted by the pathogenic bacterium *Staphylococcus aureus* (Tayeb-Fligelman et al., 2017).

**Fig. 3.**
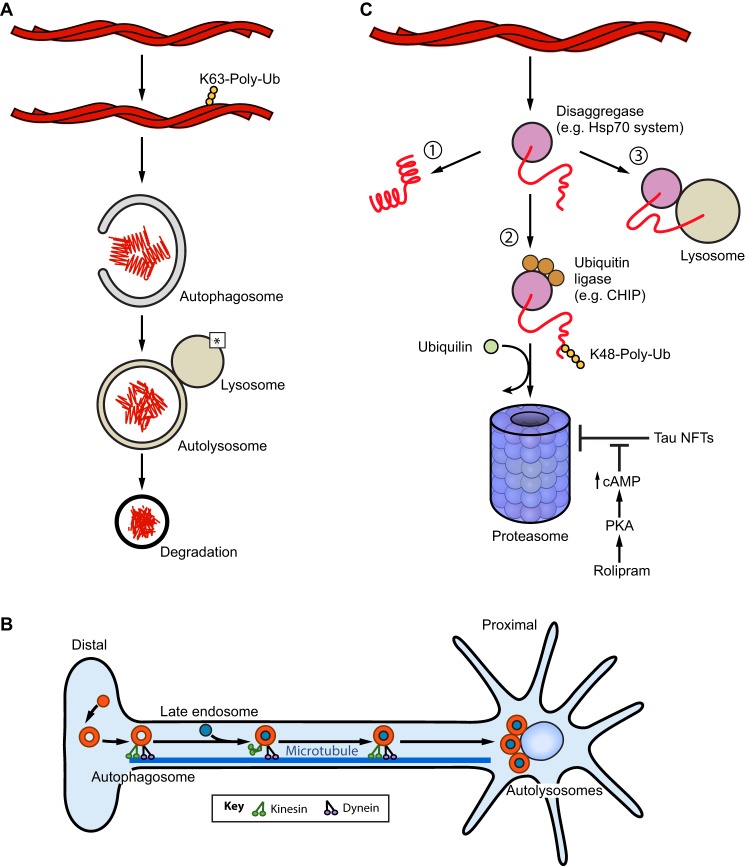
**Amyloid degradation via autophagy and the ubiquitin-proteasome system.** (A) In macroautophagy, K63 poly-ubiquitylated aggregates are engulfed by autophagosomes and targeted for degradation. Fusion of the autophagosome with a lysosome forms an autolysosome that degrades the aggregate cargo. Lysosome acidification relies on presenilin 1 (PS1), which recruits a proton pump to the lysosome that is critical for autolysosome acidification (denoted *) (Lee et al., 2010b). (B) In neurons, autophagosome formation occurs in the distal axon. Autophagosomes then fuse with late endosomes as they are retrogradely transported along microtubules by dynein toward the soma. Autophagosomes also bind kinesin motors, which must be negatively regulated to yield robust retrograde motility driven by dynein. Upon arrival in the soma, autophagosomes mature into autolysosomes via fusion with lysosomes. (C) Protein disaggregases such as Hsp70 in combination with Hsp110 and Hsp40 can extract polypeptides from aggregates and allow them to: (1) refold, (2) be degraded by the proteasome or (3) be degraded by chaperone-mediated autophagy. Polypeptides extracted from aggregates can be ubiquitylated by Hsp70-associated ubiquitin ligases such as CHIP (McDonough and Patterson, 2003). The polypeptides are then brought to the proteasome for degradation by shuttles such as UBQLN2 (Hjerpe et al., 2016). Tau fibrils can inhibit proteasome activity and this inhibition can be relieved by increasing cAMP–PKA signaling with the small-molecule Rolipram (Myeku et al., 2016). Alternatively, polypeptides may be preferentially translocated into the lysosome for degradation via a process called chaperone-mediated autophagy (Schneider and Cuervo, 2013).

**Fig. 4.**
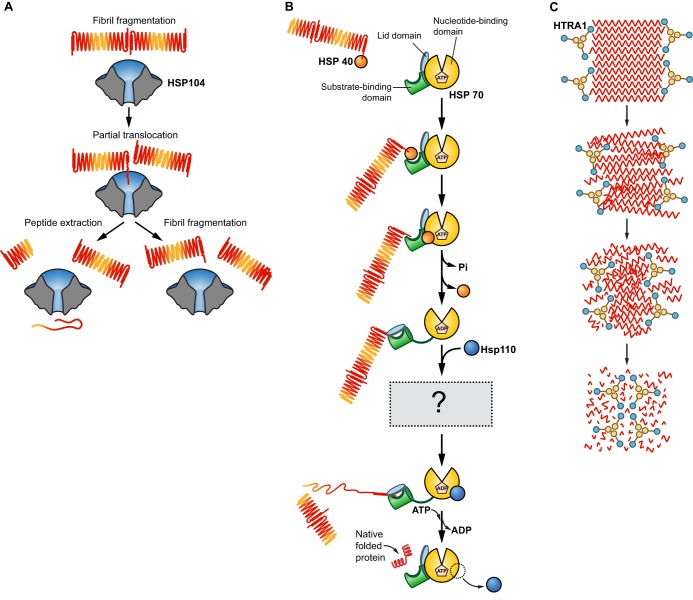
**Amyloid-disaggregase machineries.** (A) Hsp104 is an AAA+ ATPase with the ability to efficiently fragment yeast prions to allow their inheritance by daughter cells. Hsp104 can fragment amyloid fibrils by partial or full translocation of a polypeptide out of the fibril, thus creating a break point (Sweeny and Shorter, 2016). (B) Hsp70 family proteins contain a nucleotide-binding domain and a substrate-binding domain. Polypeptides trapped in fibrils are recruited to the substrate-binding domain of Hsp70 by Hsp40 family proteins. Concomitant binding of Hsp40 and substrate to Hsp70 facilitates ATP hydrolysis and a conformational change in Hsp70 to a closed state, which traps the substrate. Then through a poorly understood mechanism, in conjunction with Hsp110 family proteins, nucleotide exchange factors for Hsp70, polypeptide is extracted and refolded into its native conformation (Nillegoda and Bukau, 2015; Torrente and Shorter, 2013). This process may require Hsp110 to engage substrate and hydrolyze ATP (Mattoo et al., 2013; Scior et al., 2018; Shorter, 2011). Hsp110, Hsp70 and Hsp40 preferentially depolymerize amyloid fibrils from their ends (Duennwald et al., 2012; Gao et al., 2015). (C) Human HtrA1 is an ATP-independent serine protease that functions as a homotrimer. HtrA1 has the PDZ domain-dependent ability to disassemble Aβ and tau fibrils followed by subsequent proteolysis by its serine protease domain (Poepsel et al., 2015).

Understanding amyloid structure (Fig. 1A), the mechanisms by which amyloids form (Fig. 1B–D), and the cellular machineries that control amyloidogenesis and related toxicity (Figs 2–4) will enable development of therapeutics for several fatal diseases. In this Review, we highlight advances in our understanding of functional and pathological amyloid fibrils. In particular, we focus on amyloid structure, formation, degradation and disaggregation.

## Functional amyloid fibrils

Many proteins adopt an amyloid conformation to perform beneficial functions in a variety of organisms (Harvey et al., 2017; Hufnagel et al., 2013; Jarosz and Khurana, 2017). In humans, these include premelanosome protein (PMEL) (Fig. 2A) (Berson et al., 2003; Fowler et al., 2006; Watt et al., 2009), cytoplasmic polyadenylation element binding protein (CPEB) 3 (Fig. 2B) (Drisaldi et al., 2015; Fioriti et al., 2015; Pavlopoulos et al., 2011; Stephan et al., 2015), several polypeptides in human seminal fluid (Box 1) (Castellano and Shorter, 2012; Roan et al., 2017) and peptide hormones (Box 1) (Fig. 2C) (Maji et al., 2009). Understanding differences between functional and pathological amyloids may inform efforts to combat amyloid in disease.

Human CPEB3 is an RNA-binding protein (RBP) with an N-terminal low-complexity domain (LCD) enriched in glutamine. This region is similar to the prion domain in *Aplysia* CPEB, which enables *Aplysia* CPEB to form infectious amyloids, termed prions (Box 2) that underpin LTP (Shorter and Lindquist, 2005; Si et al., 2003a,b). CPEB3 displays prion-like behavior in yeast (Si et al., 2003b; Stephan et al., 2015). In its basal state, synaptic CPEB3 is soluble and represses translation of target mRNAs in the synaptic cytosol (Fig. 2B) (Fioriti et al., 2015). Upon neuronal stimulation (Fig. 2B, step 1), CPEB3 fibrillizes (Fig. 2B, step 2) and triggers polyadenylation and increased translation of specific transcripts essential for LTP, including AMPA receptors (Fig. 2B, step 3) (Fioriti et al., 2015). Unlike pathogenic amyloids, CPEB3 fibrillization supports synaptic plasticity, partially due to post-translational modifications that regulate its solubility (Drisaldi et al., 2015; Fioriti et al., 2015). In its basal soluble state, CPEB3 is SUMOylated, preventing its aggregation, but upon synaptic stimulation, CPEB3 is ubiquitylated and deSUMOylated, which promotes CPEB3 assembly into active fibrils (Fig. 2B, step 2) (Drisaldi et al., 2015; Pavlopoulos et al., 2011). SUMOylation can regulate amyloidogenesis by increasing protein solubility, but in other cases can promote aggregation and toxicity (Drisaldi et al., 2015; Krumova et al., 2011; Krumova and Weishaupt, 2013; Lee et al., 2013; O'Rourke et al., 2013; Rott et al., 2017).

Box 2. Prions – agents of disease or evolutionary advantageous structures? Prions are infectious proteins that typically propagate via an infectious, self-templating amyloid form (Cushman et al., 2010; Prusiner, 1998; Shorter and Lindquist, 2005). The infectious amyloid converts properly folded prion proteins into the self-templating amyloid form, thereby creating a protein-misfolding chain reaction (Aguzzi and Calella, 2009; Collinge, 2001; Prusiner, 1998; Shorter and Lindquist, 2005). Prions formed by mammalian prion protein (PrP) cause Creutzfeldt–Jakob disease (CJD), Gerstmann–Sträussler–Scheinker syndrome, fatal familial insomnia, and kuru in humans, scrapie in sheep, bovine spongiform encephalopathy (BSE) in cattle and chronic wasting disease in cervids (Prusiner, 1998; Shorter and Lindquist, 2005). Prion forms of PrP can propagate disease across individuals of different species (Prusiner, 1998; Shorter and Lindquist, 2005). New hosts have been infected by prions through contaminated blood transfusions, growth hormone and medical instruments (Aguzzi and Calella, 2009; Dormont, 1996; Peden et al., 2004). Other cases of CJD are linked to consumption of meat from cattle harboring prions that cause BSE, commonly known as mad cow disease (Dormont, 2002; Mok et al., 2017). Likewise, kuru, another human prion disease, can spread through cannibalistic consumption of infected brains (Haik and Brandel, 2014). In the context of these human diseases, prions are highly detrimental, but in other contexts prions can confer beneficial, heritable phenotypes. In yeast, Sup35 and Mot3 prions confer selective advantages in stressful and rapidly fluctuating environments (Harvey et al., 2017; Jarosz and Khurana, 2017; March et al., 2016; Shorter and Lindquist, 2005). A transcription terminator Rho of *Clostridium botulinum* might form a prion in the context of *E. coli* and yeast, although these putative Rho prions have not been reported to occur in *C. botulinum* and it is unclear whether they might be beneficial, neutral or detrimental (Yuan and Hochschild, 2017).

PMEL forms amyloid within melanin-biosynthetic organelles called melanosomes (Berson et al., 2003; Fowler et al., 2006). Within melanosomes, PMEL fibrils support organelle architecture and melanin biosynthesis, and are not inherently toxic to melanocytes (Berson et al., 2003; Fowler et al., 2006). The benign nature of PMEL amyloid results from tight spatiotemporal regulation of fibrillogenesis (Fig. 2A). PMEL fibrillization is strictly localized to sites of melanasome biogenesis, minimizing deleterious interactions with other proteins (Ho et al., 2016; Watt et al., 2013). PMEL is synthesized as an integral membrane glycoprotein in the endoplasmic reticulum, enters the secretory pathway and is post-translationally modified in the trans-Golgi network (Fig. 2A) (Ho et al., 2016; Watt et al., 2013). PMEL reaches the plasma membrane and is endocytosed before being sorted into endosomal compartments that mature into melanosomes (Fig. 2A) (Ho et al., 2016; Watt et al., 2013). Only at this stage is PMEL cleaved into a fibrillogenic fragment and released into the lumen. However, PMEL fibrillization is restricted to the luminal surface of intraluminal vesicles (Fig. 2A, stage I and II) (Ho et al., 2016; Watt et al., 2013). Formation of supramolecular structures masks the amyloidogenic core of PMEL fibrils, reducing any sequestration of nearby proteins (Fig. 2A, stage III and IV) (Fowler et al., 2006; Raposo et al., 2001). Mature PMEL fibrils promote melanin biosynthesis, a key melanosome function (Fowler et al., 2006). PMEL fibrils stack laterally, forming sheets that serve as scaffolds to concentrate melanin (Fowler et al., 2006). These PMEL functions depend on amyloid structures that assemble locally and rapidly. Thus, any toxic PMEL oligomers that might form before amyloid exist only fleetingly (Fowler et al., 2006).

Although functional amyloids may be biophysically similar to pathological amyloids, their aggregation is highly orchestrated by strict compartmentalization and post-translational processing. Many proteins that form pathological amyloids can also be regulated via these mechanisms but readily escape regulatory checks and undergo inappropriate amyloidogenesis. A striking example is the parallel between PMEL and amyloid-precursor protein (APP) processing. Both precursor proteins are expressed as membrane proteins and are cleaved into their mature forms (Benilova et al., 2012; Rochin et al., 2013; Watt et al., 2013). However, PMEL is specifically compartmentalized within melanosomes (Watt et al., 2013), whereas formation of neurotoxic amyloid-beta (Aβ) peptides (especially Aβ42 and Aβ43) is due to improper cleavage of APP by β- and γ-secretases instead of α-secretase (Benilova et al., 2012). Thus, subtle alterations in regulation can unleash devastating amyloidogenic species.

## Pathological amyloid fibrils

Although many proteins form functional amyloid, some amyloids are pathological. High thermodynamic stability and transmissibility contribute to amyloid pathogenicity (Cushman et al., 2010; Guo and Lee, 2014; Jucker and Walker, 2013; Knowles et al., 2014). Amyloids can propagate via self-templating, which converts natively folded copies of proteins to the amyloid form (Nelson et al., 2005). Amyloid stability promotes accumulation and poses a challenge to proteostasis. The mechanisms by which disease proteins aggregate and cause toxicity is not fully understood (Jucker and Walker, 2013; Knowles et al., 2014). Interestingly, in experimental and disease settings, amyloid fibrils can spread between cells within an individual, contributing to classical patterns of disease progression (Clavaguera et al., 2009; Cushman et al., 2010; de Calignon et al., 2012; Guo and Lee, 2014; Jucker and Walker, 2013; Ulusoy et al., 2013; Volpicelli-Daley et al., 2011). Furthermore, prions can spread naturally between individuals in a population (Box 2) (Choi et al., 2016; Cushman et al., 2010; Prusiner, 1998; Terry et al., 2016).

Alzheimer's disease (AD) is a common neurodegenerative disease in which Aβ assembles into insoluble amyloid fibrils that accumulate in extracellular neuritic plaques (Batarseh et al., 2016; Benilova et al., 2012; Giasson et al., 2003b; Hardy and Selkoe, 2002; Selkoe and Hardy, 2016). Accumulation of plaques is accompanied by disruption of synaptic function, neuronal atrophy of the hippocampus and cerebral cortex, dementia and cognitive impairment (Braak and Braak, 1991; Khan et al., 2014; Thal et al., 2002). Some hypothesize that Aβ fibrils or soluble Aβ oligomers are intrinsically toxic to cells, while others suggest that Aβ oligomers or fibrils enhance formation of tau tangles (Guo and Lee, 2014; He et al., 2018). Aβ fibrils are also implicated in cerebral amyloid angiopathy (CAA) where they accumulate in cerebral vasculature, causing hemorrhage, stroke and inflammation (Batarseh et al., 2016; Love et al., 2009).

Aβ is generated via cleavage of the membrane protein APP by β- and γ-secretases, creating 36–43 amino acid Aβ peptides, including amyloidogenic Aβ40, Aβ42 and Aβ43 peptides (Benilova et al., 2012; Bossy-Wetzel et al., 2004; Selkoe, 2001; Wälti et al., 2016). Normally, APP is cleaved by α- and γ-secretases into α and C83 precursor peptides, from which p3 peptides are generated (Selkoe, 2001). Pathological cleavage of APP by β-secretase occurs in sporadic AD, but missense mutations in APP such as K595N/M596L in the β-cleavage site can cause increased Aβ production and early onset AD (Benilova et al., 2012; Citron et al., 1992; Hardy and Selkoe, 2002; Selkoe and Hardy, 2016). Alternative missense mutations in APP, such as the Arctic mutation (E693G), cause reduced Aβ production but enhance Aβ protofibril formation (Benilova et al., 2012; Nilsberth et al., 2001; St George-Hyslop, 2000). Other mutations in the γ-cleavage site result in varying ratios of Aβ40, Aβ42 and Aβ43 (St George-Hyslop, 2000).

Aβ peptides exhibit differential toxicity. Aβ43 is the most cytotoxic and Aβ40 is the most benign (Benilova et al., 2012; Burnouf et al., 2015; Saito et al., 2011; Seither et al., 2014). Aβ43 fibrils confer the highest toxicity *in vivo* and enhance Aβ40 toxicity (Benilova et al., 2012; Burnouf et al., 2015; Saito et al., 2011). Aβ40 and Aβ42 fibrils adopt an S-shaped conformation of short β-strands linked by bends, forming in-register stacks of parallel cross-β subunits (Colvin et al., 2016; Tycko, 2016; Wälti et al., 2016). It is likely to be significant that the C-terminal portion of Aβ is exposed on the surface of Aβ42 fibrils but sequestered in the core of Aβ40 fibrils (Colvin et al., 2016; Tycko, 2016; Wälti et al., 2016). These differences may explain degrees of neurotoxic interactions (Bertini et al., 2011; Colvin et al., 2016; Paravastu et al., 2008; Tycko, 2016; Wälti et al., 2016). Remarkably, Aβ40 and Aβ42 form a cloud of distinct fibril structures in AD, with more rapidly progressing AD connected with more distinct Aβ40 and Aβ42 fibril structures (Lu et al., 2013; Qiang et al., 2017).

Tau, an intrinsically disordered, microtubule-binding protein, also forms amyloids linked to AD (Giasson et al., 2003b; Lee et al., 1991). Tau amyloid fibrils form intracellular neurofibrillary tangles (NFTs) in AD brains and are also found in frontotemporal dementia (FTD), Pick's disease, progressive supranuclear palsy, Parkinson's disease (PD) and dementia with Lewy bodies (DLB) (Giasson et al., 2003b; Lee et al., 1991). Mutations in the gene *MAPT* encoding tau are linked to FTD, and some of these, including P301L, V337M and R406W, accelerate tau fibrillization (Ballatore et al., 2007; Goedert and Jakes, 2005; Nacharaju et al., 1999).

The prominent protein associated with PD and DLB is α-synuclein (αSyn; encoded by *SNCA*), which is intrinsically disordered and forms amyloid inclusions called Lewy bodies (LB) (Bossy-Wetzel et al., 2004; Chu and Kordower, 2010; Giasson et al., 2003b; Spillantini et al., 1997). LBs form in diverse brain regions, conferring a spectrum of clinical manifestations. Classic PD is associated with substantia nigra pathology and motor symptoms, while pathology in DLB is in the frontal cortex and confers cognitive symptoms (Braak et al., 2003; Giasson et al., 2003b; Klein and Westenberger, 2012). Mutations in the *SNCA* gene such as A53T, E46K and A30P cause early-onset PD. A53T and E46K accelerate αSyn fibrillization, whereas A53T, E46K and A30P all accelerate formation of toxic pre-amyloid αSyn oligomers (Conway et al., 2000; Fredenburg et al., 2007; Greenbaum et al., 2005; Klein and Westenberger, 2012; Ono et al., 2011).

Huntington's disease (HD) is characterized by chorea, behavioral and psychiatric disturbances, cognitive impairment and in some cases dementia (Roos, 2010). HD affects the striatum (Roos, 2010). In HD, a CAG-repeat expansion in exon 1 of the Huntingtin gene encodes a polyglutamine (polyQ) repeat expansion in the N-terminal region of mutant huntingtin protein (Htt), accelerating amyloidogenesis (Scherzinger et al., 1997). Expansion length inversely correlates with age of HD onset in a dominant manner (Huntington's Disease Collaborative Research Group, 1993; Lee et al., 2012). Infrared microspectroscopy of Htt inclusions revealed a large degree of structural polymorphism, including amyloid inclusions in more severely affected brain regions (André et al., 2013; Nekooki-Machida et al., 2009). Remarkably, the CAG-repeat expansions also undergo repeat-associated non-ATG (RAN) translation yielding polyalanine, polyserine, polyleucine and polycysteine peptide repeats that aggregate in the brains of HD patients (Bañez-Coronel et al., 2015).

Amyotrophic lateral sclerosis (ALS) presents with progressive muscle wasting and weakness culminating in paralysis as a result of upper and lower motor neuron degeneration (Taylor et al., 2016). ALS has been linked to protein aggregates that do not always react with diagnostic amyloid dyes (Bigio et al., 2013; Furukawa et al., 2011; Kerman et al., 2010; Lee and Kim, 2015; Robberecht and Philips, 2013; Robinson et al., 2013). Mutations in superoxide dismutase 1 (SOD1) underlie ∼20% of familial ALS cases, and SOD1 mutants can form amyloid fibrils (Ivanova et al., 2014; Lee and Kim, 2015; Renton et al., 2014). Transgenic mice expressing human SOD1^H46R/H48Q^ or ALS-linked SOD1 variants G37R, G85R or G93A present with fibrillar (thioflavin-S-reactive) SOD1 inclusions (Wang et al., 2002). Additionally, SOD1 forms fibrils *in vitro* with amyloid-like characteristics (Chan et al., 2013; Chia et al., 2010; Ivanova et al., 2014). Importantly, synthetic SOD1 amyloid can propagate in neuronal cultures and induces ALS-like phenotypes in mice (Ayers et al., 2016a,b; Münch et al., 2011).

In ∼97% of ALS cases, cytoplasmic aggregates of TDP-43, a RBP with a prion-like domain (PrLD), are found in degenerating motor neurons (Guo and Shorter, 2017; Johnson et al., 2009; Ling et al., 2013; Neumann et al., 2006). Human PrLDs possess an amino acid composition similar to yeast prion domains, which are LCDs enriched in glycine and polar, uncharged amino acids including glutamine, asparagine, tyrosine and serine (Alberti et al., 2009; Harrison and Shorter, 2017; Kim et al., 2013; March et al., 2016; Shorter and Lindquist, 2005). In ALS cases without TDP-43 or SOD1 aggregates, cytoplasmic aggregates of FUS, another RBP with a PrLD, are found in degenerating neurons (Harrison and Shorter, 2017; Ling et al., 2013; March et al., 2016; Sun et al., 2011). Additional RBPs with PrLDs also aggregate in ALS, including TAF15 and EWSR1 (Couthouis et al., 2012, 2011; Harrison and Shorter, 2017).

Multisystem proteinopathy (MSP) is an inherited degenerative disorder that can affect muscle, bone and the nervous system. Two other RBPs with PrLDs, heteronuclear (hn)RNPA1 and hnRNPA2, form cytoplasmic aggregates in degenerating tissues (Kim et al., 2013). TDP-43, FUS, hnRNPA1 and hnRNPA2 are predominantly nuclear RBPs that shuttle to and from the cytoplasm but are sequestered in cytoplasmic aggregates during disease (Harrison and Shorter, 2017). These RBPs have important functions in transcription, translation, pre-mRNA splicing, RNA processing, and mRNA localization and transport (Alami et al., 2014; Colombrita et al., 2009; Kiebler and Bassell, 2006; Kim et al., 2013). Mutations in the nuclear localization sequence (NLS) of FUS promote cytoplasmic mislocalization and cause ALS (Bosco et al., 2010; Dormann et al., 2010; Kwiatkowski et al., 2009; Ling et al., 2013; Vance et al., 2009). By contrast, the majority of disease-associated mutations in TDP-43, hnRNPA1 and hnRNPA2 are located in the PrLD, which can enhance fibrillization propensity (Johnson et al., 2009; Kim et al., 2013; March et al., 2016; Shorter and Taylor, 2013).

Familial amyloid polyneuropathy (FAP) is distinguished by accumulation of amyloid deposits of transthyretin (TTR) in the peripheral nervous system (Eisele et al., 2015; Planté-Bordeneuve and Said, 2011). TTR is a stable tetrameric protein, which transports thyroid hormone, thyroxine and retinol-binding protein bound to retinol, in the serum and cerebrospinal fluid (Planté-Bordeneuve and Said, 2011). TTR also serves as a chaperone and inhibits amyloidogenesis of Aβ and microbial CsgA (Jain et al., 2017; Liu and Murphy, 2006). In FAP, mutations destabilize TTR tetramers, promoting dissociation into monomers which expose hydrophobic residues that drive rapid amyloidogenesis via downhill polymerization (DP, Fig. 1B) (Hurshman et al., 2004). TTR amyloid accumulation leads to reduced nerve fiber density and degeneration of peripheral neurons (Coelho et al., 2016).

Amyloidosis is not restricted to neurodegenerative disease. Indeed, several polypeptides, including TTR, immunoglobulin light chains and serum amyloid A, form amyloids that accumulate to debilitating tissue-damaging levels (Wechalekar et al., 2016). Furthermore, amylin is a peptide hormone secreted from pancreatic β-cells that inhibits glucagon secretion. In nearly all type II diabetes patients, amylin accumulates in amyloid deposits in the pancreas (Westermark and Westermark, 2013; Westermark et al., 2011). Amylin fibrils and pre-amyloid oligomers contribute to pancreatic β-cell degeneration in type II diabetes (Abedini et al., 2016; Cao et al., 2013; Hebda and Miranker, 2009; Krotee et al., 2017). Amyloidogenesis also occurs in cancer. Thus, p53 (also known as Tp53) can become sequestered in amyloid forms that reduce its tumor-suppression activity in cancer cells (Silva et al., 2014; Xu et al., 2011). Remarkably, rationally designed peptide-based inhibitors of p53 amyloidogenesis can rescue p53-mediated tumor suppression in ovarian carcinomas (Soragni et al., 2016).

### Amyloid assembly

Proteins can form amyloids via distinct mechanisms (Fig. 1B–D). Some amyloidogenic proteins, such as tau and αSyn, are natively unfolded (Cleveland et al., 1977; Del Mar et al., 2005; Mukrasch et al., 2009; Weinreb et al., 1996). Structural disorder exposes short segments of proteins called steric zippers that can form cross-β spines of amyloid fibrils via homotypic interdigitating interactions in parallel or antiparallel arrangements (Goldschmidt et al., 2010; Nelson et al., 2005; Rodriguez et al., 2015). While steric-zipper motifs are a common feature of proteins, they are generally positioned in folded regions and are therefore unavailable for amyloidogenic interactions (Goldschmidt et al., 2010). Many proteins have a single intrinsically unfolded domain (e.g. a PrLD), which can drive amyloidogenesis while the rest of the protein remains correctly folded (King et al., 2012; Li et al., 2013). Additionally, mutations in unfolded domains can introduce potent steric zippers that accelerate fibrillization observed in diseases, such as MSP-linked hnRNPA1^D262V^ and hnRNPA2^D290V^ (Kim et al., 2013; Molliex et al., 2015; Shorter and Taylor, 2013). Indeed, in these cases the disease mutation likely shifts fibrillization to a pathological zipper-based mechanism and away from a low-complexity, aromatic-rich, kinked segment (LARK)-based mechanism that may underpin biogenesis of membraneless organelles (Hughes et al., 2018). Glutamine repeat expansions readily form cross-β structures, as seen with polyQ expansions in Htt or in the ataxin 1 PrLD (Banfi et al., 1994; March et al., 2016; Perutz et al., 1994; Scherzinger et al., 1997).

Mutations are not necessary for amyloidogenicity. In sporadic disease, it is often wild-type protein that fibrillizes (e.g. tau in AD, αSyn in PD and TDP-43 in ALS). Any protein can probably form amyloid under specific environmental conditions (Fändrich and Dobson, 2002; Fändrich et al., 2001, 2003). Even structured proteins, such as TTR, can spontaneously transition between folding states capable of fibrillization (Colon and Kelly, 1992; Hurshman et al., 2004). Unfolded states may be accessed under stressful conditions including heat or denaturation (Booth et al., 1997; Colon and Kelly, 1992; Eisele et al., 2015; Kelly, 1998). Alternatively, intrinsically unfolded domains of wild-type proteins can be exposed after proteolysis, as in Aβ processing (Benilova et al., 2012; Hardy and Selkoe, 2002; Selkoe and Hardy, 2016). Furthermore, many proteins are intrinsically unfolded but do not form amyloid (Dunker et al., 2008), indicating that unfolding is necessary but not sufficient for amyloidogenesis. Indeed, amyloidogenic motifs are ubiquitous, yet cells are generally effective at preventing aggregation due to proteostasis networks (Balch et al., 2008).

Two distinct mechanisms can underpin amyloid assembly: DP and nucleated conformational conversion (NCC) (Eisele et al., 2015). The precise mechanism employed depends on the specific protein. In DP, the rate-limiting step is dissociation of stable, native oligomers into amyloidogenic monomers that rapidly fibrillize (Fig. 1B) (Eisele et al., 2015; Hurshman et al., 2004; Lai et al., 1996). Here, the lag phase of assembly is dictated by slow disassembly of native oligomers (Eisele et al., 2015). FAP-linked mutations in TTR destabilize the native tetramer and facilitate formation of amyloidogenic monomers (Fig. 1B) (Eisele et al., 2015; Hammarstrom et al., 2003). Typically, amyloids that form via DP show poor seeding activity (Fig. 1B), which may preclude efficient transmissibility of the amyloid phenotype (Castellano et al., 2015b; Eisele et al., 2015; Hurshman et al., 2004; Lai et al., 1996; Westermark and Westermark, 2008). Indeed, amyloid with poor seeding activity is unlikely to exhibit prion behavior (Box 2).

NCC is a variation of earlier nucleated polymerization models (Jarrett and Lansbury, 1993), but more accurately explains the sigmoidal kinetics and concentration dependence of spontaneous amyloidogenesis (Lee et al., 2011b; Serio et al., 2000; Shorter and Lindquist, 2004). In NCC, soluble monomers are initially in equilibrium with molten soluble oligomers (Fig. 1C) (Scheibel and Lindquist, 2001; Serio et al., 2000). During the lag phase of assembly, these molten soluble oligomers gradually rearrange into amyloidogenic oligomers, which rapidly form cross-β nuclei, ending the lag phase (Fig. 1C) (Krishnan et al., 2012; Krishnan and Lindquist, 2005; Scheibel and Lindquist, 2001; Serio et al., 2000; Shorter and Lindquist, 2004, 2005). Once cross-β nuclei have formed, fibrillization proceeds rapidly as nuclei recruit and convert soluble monomers (and molten soluble oligomers) into the cross-β form at growing fibril ends (Fig. 1C) (Krishnan et al., 2012; Krishnan and Lindquist, 2005; Scheibel et al., 2004; Serio et al., 2000; Shorter and Lindquist, 2004). Preformed fibrils abolish the lag phase of amyloid formation via immediate templating of the amyloid conformation (Fig. 1C) (Lee et al., 2011b; Serio et al., 2000; Shorter and Lindquist, 2005). This seeding mechanism enables amyloids to convert non-amyloid copies of the protein to the amyloid state and contributes to transmission of phenotypes encoded by amyloid (Shorter, 2010; Shorter and Lindquist, 2005).

Typically, self-templating by an amyloid is highly specific due to primary-sequence-enforced structural constraints (Del Mar et al., 2005; Riek and Eisenberg, 2016). Thus, other copies of the same protein are efficiently converted into the amyloid form. Rarely, amyloid forms of one protein can ‘cross-seed’ fibrillization of another protein. Specifically, αSyn can promote tau fibrillization (Giasson et al., 2003a), and Rnq1 prions cross-seed polymerization of Sup35 prions (Derkatch et al., 2004; Duennwald et al., 2012). Cross-seeding tends to be inefficient and self-seeding predominates once an amyloid has been nucleated (Derkatch et al., 2004).

High local concentrations of unfolded LCDs of proteins can drive liquid–liquid phase-separation (LLPS), which underpins formation of membraneless organelles, including stress granules (SGs) and nucleoli (Brangwynne, 2013; Brangwynne et al., 2015; Feric et al., 2016; Franzmann et al., 2018; March et al., 2016; Nott et al., 2016, 2015; Shin and Brangwynne, 2017; Shorter, 2016b; Zhu and Brangwynne, 2015). LLPS is driven by transient, weak intermolecular associations of PrLDs and other domains within RBPs such as hnRNPA1, TDP-43 or FUS (Fig. 1D) (Burke et al., 2015; Conicella et al., 2016; Lin et al., 2015; Monahan et al., 2017; Shorter, 2017b). In the liquid state, interactions between PrLDs are labile, perhaps even including transient cross-β interactions (Molliex et al., 2015; Murakami et al., 2015; Murray et al., 2017; Patel et al., 2015). However, if these RBPs persist in the condensed phase-separated liquid state, they eventually form stable hydrogels and pathological fibrils, in a manner akin to NCC but on a macroscopic scale (Fig. 1D) (Guo and Shorter, 2015; Kato et al., 2012; Kato and McKnight, 2017; Lin et al., 2015; Molliex et al., 2015; Murakami et al., 2015; Patel et al., 2015; Shin et al., 2017). Remarkably, ALS-linked mutations in the PrLD of hnRNPA1 and FUS accelerate transitions from liquid to gel states, which likely accelerates disease (Molliex et al., 2015; Patel et al., 2015).

How do mature amyloid fibrils affect the levels of toxic soluble oligomers? While the answer to this question is debated, kinetic analysis of Aβ42 fibrillization suggests that there is a secondary nucleation mechanism: at critical concentrations, the lateral face of amyloid fibrils catalyzes assembly of monomeric peptides or molten oligomers into toxic, soluble oligomers (Fig. 1C) (Cohen et al., 2013). Lateral fibril surfaces act as a template against which monomers or molten oligomers can rapidly morph into amyloidogenic oligomers. These amyloidogenic oligomers then detach and mature into their own fibrils, contributing to a vicious feedforward loop of rapid amyloid assembly (Cohen et al., 2013). Combining facets of NCC, secondary nucleation events and infrequent fibril fragmentation provides enough degrees of freedom to accurately describe amyloid assembly kinetics (Cohen et al., 2013; Knowles et al., 2009, 2014).

Understanding which steps are critical in amyloidogenesis provides insight for interventions. In NCC, agents that prevent the transition from molten oligomers to amyloidogenic nuclei could be valuable, such as the tea polyphenol EGCG, which promotes formation of non-toxic, off-pathway oligomers (Bieschke et al., 2010; Eisele et al., 2015; Roberts et al., 2009; Roberts and Shorter, 2008). Likewise, NCC by Sup35 and Aβ42 is inhibited by the small-molecule DAPH-12, which abrogates maturation of molten oligomers into amyloidogenic oligomers (Wang et al., 2008).

Amyloid fibrils are sufficient to encode disease. Thus, introduction of synthetic PrP amyloids into mice induces prion disease (Choi et al., 2016; Colby et al., 2009; Legname et al., 2004, 2006; Wang et al., 2010), whereas αSyn fibrils induce a PD-like disease (Luk et al., 2012). However, while amyloid fibrils are sufficient to cause neurodegeneration (Choi et al., 2016; Luk et al., 2012), soluble misfolded oligomers might be the most toxic species (Bucciantini et al., 2002; Conway et al., 2000; Kayed et al., 2003; Martin et al., 2012). It is now clear that misfolded oligomers and amyloid fibrils are inextricably linked, as these oligomers form on the lateral faces of fibrils (Buell et al., 2014; Cohen et al., 2013; Meisl et al., 2014). Thus, wherever there is amyloid, there are likely to be toxic oligomers. Proteins can gain toxic function in the misfolded state, as with SOD1 (Bruijn et al., 1998) or FUS (Sharma et al., 2016), but proteins can also lose functionality upon misfolding. This loss of function may be particularly important for toxicity when essential proteins, such as TDP-43, become sequestered in mislocalized aggregated states (Guo and Shorter, 2017).

Kinetic stabilization of polypeptides in their native states can prevent amyloidogenesis (Hammarstrom et al., 2001). This strategy is particularly attractive if the native state has a defined architecture that can be stabilized by small molecules (Hammarstrom et al., 2003). Indeed, TTR amyloidogenesis can be diminished by the small molecule tafamidis, which stabilizes mutant TTR in its native tetrameric form (Fig. 1B) (Bulawa et al., 2012; Cho et al., 2015; Coelho et al., 2013, 2016). Tafamidis is an approved and effective FAP treatment in Europe, Japan, Brazil, Argentina, Mexico and Israel (but bafflingly not yet in the USA). Tafamidis reduces TTR amyloid and soluble misfolded TTR assemblies in FAP (Barroso et al., 2017; Coelho et al., 2012; Schonhoft et al., 2017), and remains the only therapeutic for a neurodegenerative disease that specifically targets the underlying causative amyloidogenesis. In a similar vein, small molecules that stabilize α-crystallin, which prevent and reverse amyloidogenesis, are exciting leads to treat cataracts (Makley et al., 2015).

## Amyloid structure

The fibrillar nature of amyloid has made its structure challenging to solve at atomic resolution but important advances have been made (Eisenberg and Sawaya, 2017; Riek and Eisenberg, 2016). The self-complementary β-strands of amyloid align orthogonally to the longitudinal fibril axis (Fig. 1A), generating the cross-β quaternary structure (Nelson et al., 2005; Riek and Eisenberg, 2016; Sunde et al., 1997). While variability exists between amyloids formed by different proteins (Fig. 1A), some common features include: β-strands maintained by steric zippers involving hydrophobic side chains or uncharged polar residues, glutamine ladders along the fibril axis, and hydrophobic packing of methyl-bearing and aromatic residues (Makin et al., 2005; Nelson et al., 2005; Riek and Eisenberg, 2016). Variability in how β-strands align exists among fibrils formed by different proteins. Amyloid β-sheets can align in parallel (Benzinger et al., 1998; Nelson et al., 2005; Petkova et al., 2002). However, anti-parallel amyloid β-sheets can also form (Qiang et al., 2012; Tycko et al., 2009).

Amyloids can also be comprised of parallel β-helices (Tsai et al., 2006). For example, HET-s forms functional prions in *Podospora anserina* (Riek and Saupe, 2016). The HET-s prion-forming domain assembles into parallel β-sheets that stack into a left-handed β-solenoid arrangement (Wasmer et al., 2008). Sup35 prions and PrP prions may also adopt β-helical structures (Govaerts et al., 2004; Krishnan and Lindquist, 2005; Wille et al., 2009). Remarkably, cryo-electron microscopy (cryo-EM) structures of tau fibrils from an AD patient reveal a combination of classic β-strand stacking and β-helical structure (Fig. 1A) (Fitzpatrick et al., 2017). The structures of paired helical filaments and straight filaments revealed differences in inter-protofilament packing that confer ultrastructural polymorphism (Fig. 1A) (Fitzpatrick et al., 2017). Solid-state NMR analysis of pathogenic αSyn fibrils revealed a glycine-rich amyloidogenic core with an arrangement resembling a Greek key (Fig. 1A) (Tuttle et al., 2016). Remarkably, amyloid fibrils formed by a portion of the PrLD of FUS also adopt a Greek key arrangement akin to pathogenic αSyn fibrils (Murray et al., 2017). Likewise, LARKs stack into kinked β-sheets that pair into protofilaments (Hughes et al., 2018).

A single protein can form different cross-β structures, termed ‘strains’. The concept of different strains encoding different phenotypes is well established for yeast and mammalian prions (Colby et al., 2009; Legname et al., 2006; Shorter, 2010; Tanaka et al., 2004). Interestingly, phenotypic severity is determined, at least partially, by optimal frangibility of a particular fibril strain. This means that the rate of fibril fragmentation, which liberates new growing fibril ends, and thus, seed formation and propagation, is an important factor in determining the strength of prion phenotypes (Colby et al., 2009; Cushman et al., 2010; Legname et al., 2006; Shorter, 2010; Tanaka et al., 2006).

Human disease amyloids also exhibit strain variation that results in disease heterogeneity (Guo et al., 2013; Lu et al., 2013; Peelaerts et al., 2015; Qiang et al., 2017; Rodriguez et al., 2015). Some disease-associated proteins may have multiple steric zippers in unfolded regions, conferring multiple points of contact and thus, variations in amyloid structure (Krotee et al., 2017; Tuttle et al., 2016). Distinct strains of Aβ and αSyn fibrils differ in structure, toxicity and propagation capability (Bousset et al., 2013; Brundin and Melki, 2017; Guo et al., 2013; King et al., 2012; Lu et al., 2013; Peelaerts et al., 2015; Qiang et al., 2017; Rodriguez et al., 2015). Differing Aβ and αSyn strains form *in vitro* under different environmental conditions (Peelaerts et al., 2015; Petkova et al., 2005; Qiang et al., 2017). Aβ fibrils found in patients presenting with AD can be polymorphic, with more aggressive forms of AD harboring a more diverse cloud of structures (Lu et al., 2013; Petkova et al., 2005; Qiang et al., 2017). Individual strains selectively seed and propagate the same strain conformation *in vitro* (Peelaerts et al., 2015; Petkova et al., 2005; Qiang et al., 2017). Furthermore, strains have differing cytotoxicity (Peelaerts et al., 2015). Taken together, these findings suggest a mechanism by which the same protein may underlie diseases with distinct clinical symptoms.

## Amyloid degradation via autophagy and the ubiquitin proteasome system

Several avenues are being explored to mitigate or reverse amyloid toxicity, including stimulating existing degradation machineries to promote clearance of toxic amyloid and oligomers (Guo et al., 2014; Wang and Saunders, 2014). Two major intracellular degradation pathways that may be bolstered therapeutically are autophagy and the ubiquitin-proteasome system (UPS) (Fig. 3A–C) (Cho et al., 2014; Ciechanover and Kwon, 2015; Victoria and Zurzolo, 2015).

Autophagy is an important degradation pathway for many disease-associated aggregates (Fig. 3A), such as those formed by TDP-43 (Barmada et al., 2014), αSyn (Webb et al., 2003), polyglutamine (Yamamoto et al., 2006), tau (Falcon et al., 2017) and Aβ (Cho et al., 2014). Proteins can undergo chaperone-mediated autophagy, where molecular chaperones deliver proteins to the lysosome for degradation (Fig. 3C) (Schneider and Cuervo, 2013), or macroautophagy, where they are enveloped within an autophagosome for delivery to the lysosome through membrane fusion (Fig. 3A,B) (Kulkarni and Maday, 2018; Maday, 2016). In neurons, macroautophagy is spatiotemporally organized such that autophagosomes form distally and transport cargo along the axon for delivery to lysosomes in the soma (Fig. 3B) (Maday and Holzbaur, 2012, 2014, 2016; Maday et al., 2012). Defects in autophagy, such as impairments of scaffolding proteins involved in autophagosome transport, or improper lysosomal acidification, are implicated in HD and AD and can cause neurodegeneration (Filimonenko et al., 2010; Hara et al., 2006; Lee et al., 2010b; Wong and Holzbaur, 2015). Thus, stimulating autophagy may reduce amyloid toxicity. Indeed, small-molecule compounds that stimulate autophagy improve TDP-43 clearance, reduce aggregates and increase survival in a neuronal model of ALS (Barmada et al., 2014). However, circumspection is needed in the development of autophagy activators, as excessive autophagy might degrade essential cell components or confer toxicity, accelerating disease progression (Yamamoto and Simonsen, 2011; Zhang et al., 2011). Nonetheless, transient or intermittent activation of autophagy warrants further investigation as a therapeutic strategy.

The UPS is another pathway critical to the degradation of many proteins, and defects in its activity have been implicated in neurodegeneration (Fig. 3C) (Ciechanover and Brundin, 2003). Thus, stimulating machinery that delivers misfolded proteins to the proteasome (Guo et al., 2014) or stimulating proteasome activity itself (Leestemaker et al., 2017) could be therapeutic. Indeed, inhibition of the deubiquitylating enzyme Usp14 enhances degradation of toxic proteins by the UPS (Homma et al., 2015; Lee et al., 2010a). A relationship between aggregated tau and proteasomal dysfunction has also been identified (Myeku et al., 2016). Tau aggregates associate with the proteasome, inhibiting its ATPase and proteolytic activities (Myeku et al., 2016). This defect is relieved by increasing cAMP–protein kinase A (PKA) signaling with Rolipram, a small molecule that increases cAMP levels by inhibiting its degradation (Fig. 3C). Rolipram restores proteasome function, decreases tau aggregate burden and improves cognitive function in mice exhibiting early-stage tauopathy (Myeku et al., 2016). Increasing proteasomal activity in the early-stage model eliminates toxic oligomers or small fibrils that seed amyloid propagation in neighboring cells, thereby inhibiting disease progression (Myeku et al., 2016). However, Rolipram was ineffective against late-stage tauopathy (Myeku et al., 2016).

## Amyloid-disaggregase machineries

Molecular chaperones and protein disaggregases maintain proteostasis (Mack and Shorter, 2016). Many chaperones such as those of the Hsp70 and Hsp90 families bind nascent and unfolded proteins under stress (Mack and Shorter, 2016). Hsp70 and Hsp90 proteins assist in protein folding by protecting exposed hydrophobic regions from aggregation (Mack and Shorter, 2016). Thus, chaperones are important inhibitors of amyloid formation (Lindberg et al., 2015). Protein disaggregases can safely reverse formation of toxic soluble misfolded oligomers and amyloid fibrils, reducing toxic species and restoring native function to proteins sequestered within aggregates (Mack and Shorter, 2016). Thus, protein disaggregases present a promising therapeutic strategy to combat both gain- and loss-of-function toxicity (Shorter, 2008, 2016a, 2017a; Vashist et al., 2010).

Yeast Hsp104 is among the most effective protein disaggregases. Hsp104 is a 102 kDa member of the AAA+ ATPase family with two nucleotide-binding domains (Sweeny and Shorter, 2016). Six protomers of Hsp104 form an offset hexameric barrel that hydrolyzes ATP to translocate polypeptides through its central pore and generate the force required for disaggregating proteins and prions (Gates et al., 2017; Sweeny et al., 2015; Yokom et al., 2016). Hsp104 disaggregates disordered aggregates, toxic soluble oligomers, yeast prions formed by Sup35, Ure2 and Rnq1, and disease-linked amyloid formed by αSyn, polyQ, tau, Aβ, PrP and amylin (Fig. 4A) (DeSantis et al., 2012; DeSantis and Shorter, 2012; Liu et al., 2011). Despite having no metazoan homolog, Hsp104 robustly rescues toxicity conferred by polyQ in worms, flies and rodents, and αSyn^A30P^ in rats (Cushman-Nick et al., 2013; Lo Bianco et al., 2008; Perrin et al., 2007; Satyal et al., 2000; Vacher et al., 2005). Engineered, potentiated variants of Hsp104 exhibit enhanced disaggregase activity that more effectively dissolves preformed αSyn, FUS and TDP-43 fibrils (Jackrel et al., 2014). Potentiated Hsp104 variants rescue FUS, TDP-43 and αSyn toxicity in yeast, reverse FUS aggregation in mammalian cells, and mitigate αSyn-induced dopaminergic neurodegeneration in *C. elegans* (Jackrel et al., 2014; Torrente et al., 2016; Yasuda et al., 2017). Engineering substrate-specific enhanced variants of Hsp104 will empower selective disaggregation of specific disease substrates to treat amyloidoses and help avoid potential off-target effects (Jackrel and Shorter, 2017).

Metazoa lack Hsp104 but are equipped with molecular chaperones capable of protein disaggregation (Nillegoda and Bukau, 2015; Shorter, 2011; Torrente and Shorter, 2013). The metazoan disaggregase system is composed of members of the Hsp110, Hsp70, Hsp40 and small heat-shock protein families (Fig. 4B) (Duennwald et al., 2012; Mattoo et al., 2013; Nillegoda and Bukau, 2015; Nillegoda et al., 2015, 2017; Rampelt et al., 2012; Shorter, 2011; Torrente and Shorter, 2013). The metazoan disaggregase system disaggregates disordered aggregates as well as Sup35, αSyn and polyQ fibrils (Duennwald et al., 2012; Gao et al., 2015; Scior et al., 2018; Shorter, 2011). Hsp110, Hsp70 and Hsp40 disassemble SGs in yeast and humans, implicating their potential for disaggregating ALS-linked proteins that colocalize with SGs (Cherkasov et al., 2013; Kedersha et al., 1999; Kroschwald et al., 2015; Shorter and Taylor, 2013; Walters et al., 2015; Walters and Parker, 2015). Hsp70 suppresses αSyn toxicity in neuroglioma cells, and pharmacological inhibition, by means of MAL3-101, or enhancement, by means of 115-7c, of Hsp70 increases or decreases toxicity, respectively (Kilpatrick et al., 2013). The small molecule YM-01 also enhances Hsp70-mediated proteasomal degradation of polyQ and tau (Abisambra et al., 2013; Wang et al. 2013). Thus, small-molecule modulation of Hsp70 may therapeutically enhance the metazoan disaggregase system in patients.

HtrA1 is a trimeric ATP-independent serine protease with one C-terminal PDZ domain that has been implicated in substrate recognition and processing (Fig. 4C) (Clausen et al., 2011; Hansen and Hilgenfeld, 2013; Truebestein et al., 2011). HtrA1 colocalizes with Aβ and tau aggregates in AD patient samples, and disassembles and degrades Aβ and tau fibrils (Fig. 4C) (Poepsel et al., 2015; Tennstaedt et al., 2012). HtrA1 may also degrade TFFBI amyloid in corneal dystrophy (Karring et al., 2012). Metalloporphyrin-induced oligomerization of HtrA1 enhances its proteolytic activity (Jo et al., 2014). Small molecules that enhance amyloid clearance by HtrA1 may be valuable therapeutics for several neurodegenerative diseases.

Therapeutics capable of upregulating amyloid degradation and disaggregation could synergize in combating amyloidoses. Aging is correlated with the accumulation of protein aggregates and with the decline in chaperone expression, autophagy and proteasome activity (Bohnert and Kenyon, 2017; Gutsmann-Conrad et al., 1998; Kaushik and Cuervo, 2015). Thus, pharmacological enhancement of disaggregation, autophagy and proteasome activity could combat age-related deterioration of proteostasis. Targeting multiple pathways simultaneously is possible. Indeed, Hsp70 can buffer toxicity, facilitate ubiquitylation and degradation of misfolded proteins, and drive protein disaggregation (Figs 3C and 4B) (Abisambra et al., 2013; Auluck et al., 2002; Ebrahimi-Fakhari et al., 2013; Jinwal et al., 2013; Kilpatrick et al., 2013; Mack and Shorter, 2016; Nillegoda and Bukau, 2015; Torrente and Shorter, 2013; Warrick et al., 1999). Therefore, upregulation of Hsp70 expression or activity could stimulate multiple pathways to combat disease (Bonini, 2002). Indeed, delivery of Hsp70 as a therapeutic has shown promise for several disorders in preclinical studies (Auluck et al., 2002; Bobkova et al., 2015; Gehrig et al., 2012; Gifondorwa et al., 2007; Kirkegaard et al., 2016, 2010; Warrick et al., 1999).

## Conclusion

In this Review, we have summarized several aspects of our understanding of amyloid structure, formation and toxicity. We have contextualized groundbreaking discoveries, and introduced several therapeutic strategies that are being explored. These illuminating advances have enhanced our understanding of amyloid, and illustrate challenges in the treatment of neurodegenerative diseases. However, much remains unknown. Many aspects of amyloidoses are highly nuanced, such as the distinction between different amyloid strains of the same protein conferring different disease phenotypes. Acquiring a deeper understanding of how amyloid is formed, disaggregated and degraded has yielded important insights and will continue to inspire new therapeutics.
